# Dynamical anchoring of distant arrhythmia sources by fibrotic regions via restructuring of the activation pattern

**DOI:** 10.1371/journal.pcbi.1006637

**Published:** 2018-12-20

**Authors:** Nele Vandersickel, Masaya Watanabe, Qian Tao, Jan Fostier, Katja Zeppenfeld, Alexander V. Panfilov

**Affiliations:** 1 Department of Physics and Astronomy, Ghent University, Belgium; 2 Department of Cardiology, Leiden University Medical Center, Leiden, the Netherlands; 3 Department of Radiology, Division of Image Processing, Leiden University Medical Centre, Leiden, the Netherlands; 4 Department of Information Technology (INTEC), IDLab, Ghent University — imec, Ghent, Belgium; 5 Laboratory of Computational Biology and Medicine, Ural Federal University, Ekaterinburg, Russia; King’s College London, UNITED KINGDOM

## Abstract

Rotors are functional reentry sources identified in clinically relevant cardiac arrhythmias, such as ventricular and atrial fibrillation. Ablation targeting rotor sites has resulted in arrhythmia termination. Recent clinical, experimental and modelling studies demonstrate that rotors are often anchored around fibrotic scars or regions with increased fibrosis. However, the mechanisms leading to abundance of rotors at these locations are not clear. The current study explores the hypothesis whether fibrotic scars just serve as anchoring sites for the rotors or whether there are other active processes which drive the rotors to these fibrotic regions. Rotors were induced at different distances from fibrotic scars of various sizes and degree of fibrosis. Simulations were performed in a 2D model of human ventricular tissue and in a patient-specific model of the left ventricle of a patient with remote myocardial infarction. In both the 2D and the patient-specific model we found that without fibrotic scars, the rotors were stable at the site of their initiation. However, in the presence of a scar, rotors were eventually dynamically anchored from large distances by the fibrotic scar via a process of dynamical reorganization of the excitation pattern. This process coalesces with a change from polymorphic to monomorphic ventricular tachycardia.

## Author summary

Rotors are waves of cardiac excitation like a tornado causing cardiac arrhythmia. Recent research shows that they are found in ventricular and atrial fibrillation. Burning (via ablation) the site of a rotor can result in the termination of the arrhythmia. Recent studies showed that rotors are often anchored to regions surrounding scar tissue, where part of the tissue still survived called fibrotic tissue. However, it is unclear why these rotors anchor to these locations. Therefore, in this work, we investigated why rotors are so abundant in fibrotic tissue with the help of computer simulations. We performed simulations in a 2D model of human ventricular tissue and in a patient-specific model of a patient with an infarction. We found that even when rotors are initially at large distances from the fibrotic region, they are attracted by this region, to finally end up at the fibrotic tissue. We called this process dynamical anchoring and explained how the process works.

## Introduction

Many clinically relevant cardiac arrhythmias are conjectured to be organized by rotors. A rotor is an extension of the concept of a reentrant source of excitation into two or three dimensions with an area of functional block in its center, referred to as the core. Rapid and complex reentry arrhythmias such as atrial fibrillation (AF) and ventricular fibrillation (VF) are thought to be driven by single or multiple rotors. A clinical study by Narayan et al. [1] indicated that localized rotors were present in 68% of cases of sustained AF. Rotors (phase singularities) were also found in VF induced by burst pacing in patients undergoing cardiac surgery [2, 3] and in VF induced in patients undergoing ablation procedures for ventricular arrhythmias [4]. Intramural rotors were also reported in early phase of VF in the human Langendorff perfused hearts [5, 6]. It was also demonstrated that in most cases rotors originate and stabilize in specific locations [4–8].

A main mechanism of rotor stabilization at a particular site in cardiac tissue was proposed in the seminal paper from the group of Jalife [9]. It was observed that rotors can anchor and exhibit a stable rotation around small arteries or bands of connective tissue. Later, it was experimentally demonstrated that rotors in atrial fibrillation in a sheep heart can anchor in regions of large spatial gradients in wall thickness [10]. A recent study of AF in the right atrium of the explanted human heart [11] revealed that rotors were anchored by 3D micro-anatomic tracks formed by atrial pectinate muscles and characterized by increased interstitial fibrosis. The relation of fibrosis and anchoring in atrial fibrillation was also demonstrated in several other experimental and numerical studies [8, 11–14]. Initiation and anchoring of rotors in regions with increased intramural fibrosis and fibrotic scars was also observed in ventricles [5, 7, 15]. One of the reasons for rotors to be present at the fibrotic scar locations is that the rotors can be initiated at the scars (see e.g. [7, 15]) and therefore they can easily anchor at the surrounding scar tissue. However, rotors can also be generated due to different mechanisms, such as triggered activity [16], heterogeneity in the refractory period [16, 17], local neurotransmitter release [18, 19] etc. What will be the effect of the presence of the scar on rotors in that situation, do fibrotic areas (scars) actively affect rotor dynamics even if they are initially located at some distance from them? In view of the multiple observations on correlation of anchoring sites of the rotors with fibrotic tissue this question translates to the following: is this anchoring just a passive probabilistic process, or do fibrotic areas (scars) actively affect the rotor dynamics leading to this anchoring? Answering these questions in experimental and clinical research is challenging as it requires systematic reproducible studies of rotors in a controlled environment with various types of anchoring sites. Therefore alternative methods, such as realistic computer modeling of the anchoring phenomenon, which has been extremely helpful in prior studies, are of great interest.

The aim of this study is therefore to investigate the processes leading to anchoring of rotors to fibrotic areas. Our hypothesis is that a fibrotic scar actively affects the rotor dynamics leading to its anchoring. To show that, we first performed a generic in-silico study on rotor dynamics in conditions where the rotor was initiated at different distances from fibrotic scars with different properties. We found that in most cases, scars actively affect the rotor dynamics via a dynamical reorganization of the excitation pattern leading to the anchoring of rotors. This turned out to be a robust process working for rotors located even at distances more than 10 cm from the scar region. We then confirmed this phenomenon in a patient-specific model of the left ventricle from a patient with remote myocardial infarction (MI) and compared the properties of this process with clinical ECG recordings obtained during induction of a ventricular arrhythmia.

## Materials and methods

### Magnetic resonance imaging

Our anatomical model is based on an individual heart of a post-MI patient reconstructed from late gadolinium enhanced (LGE) magnetic resonance imaging (MRI) was described in detail previously [20]. Briefly, a 1.5T Gyroscan ACS-NT/Intera MR system (Philips Medical Systems, Best, the Netherlands) system was used with standardized cardiac MR imaging protocol. The contrast –gadolinium (Magnevist, Schering, Berlin, Germany) (0.15 mmol/kg)– was injected 15 min before acquisition of the LGE sequences. Images were acquired with 24 levels in short-axis view after 600–700 ms of the R-wave on the ECG within 1 or 2 breath holds. The in-plane image resolution is 1 mm and through-plane image resolution is 5 mm. Segmentation of the contours for the endocardium and the epicardium was performed semi-automatically on the short-axis views using the MASS software (Research version 2014, Leiden University Medical Centre, Leiden, the Netherlands).

The myocardial scar was identified based on signal intensity (SI) values using a validated algorithm as described by Roes et al. [21]. In accordance with the algorithm, the core necrotic scar is defined as a region with SI >41% of the maximal SI. Regions with lower SI values were considered as border zone areas. In these regions, we assigned the fibrosis percentage as normalized values of the SI as in Vigmond et al. [22]. In the current paper, fibrosis was introduced by generating a random number between 0 and 1 for each grid point and if the random number was less than the normalized SI at the corresponding pixel the grid point was considered as fibroblast.

Currently there is no consensus on how the SI values should be used for clinical assessment of myocardial fibrosis and various methods have been reported to produce significantly different results [23]. However, the method from Vigmond et al. properly describes the location of the necrotic scar region in our model as for the fibrosis percentage of more than 41% we observe a complete block of propagation inside the scar. This means that all tissue which has a fibrotic level higher than 41% behaves like necrotic scar.

### Electrophysiology model

The approach and the 2D model was described in detail in previous work [24–26]. Briefly, for ventricular cardiomyocyte we used the ten Tusscher and Panfilov (TP06) model [27, 28], and the cardiac tissue was modeled as a rectangular grid of 1024 × 512 nodes. Each node represented a cell that occupied an area of 250 × 250 *μ*m^2^. The equations for the transmembrane voltage are given by

$$\begin{array}{c} C_{m}\frac{dV_{ik}}{dt}=\sum_{\alpha ,\beta \in \{-1,+1\}}\eta_{ik}^{\alpha \beta }g_{\text{gap}}(V_{i+\alpha ,k+\beta }-V_{ik})-I_{\text{ion}}\left(V_{ik},\ldots \right), \end{array}$$ (1)

where *V*_*ik*_ is the transmembrane voltage at the (*i*, *k*) computational node, *C*_*m*_ is membrane capacitance, *g*_gap_ is the conductance of the gap junctions connecting two neighboring myocytes, *I*_ion_ is the sum of all ionic currents and $\eta_{ik}^{\alpha \beta }$ is the connectivity tensor whose elements are either one or zero depending on whether neighboring cells are coupled or not. Conductance of the gap junctions *g*_gap_ was taken to be 103.6 nS, which results in a maximum velocity planar wave propagation in the absence of fibrotic tissue of 72 cm/s at a stimulation frequency of 1 Hz. *g*_gap_ was not modified in the fibrotic areas. A similar system of differential equations was used for the 3D computations where instead of the 2D connectivity tensor $\eta_{ik}^{\alpha \beta }$ we used a 3D weights tensor $w_{ijk}^{\alpha \beta \gamma }$ whose elements were in between 0 and 1, depending both on coupling of the neighbor cells and anisotropy due to fiber orientation. Each node in the 3D model represented a cell of the size of 250 × 250 × 250 *μ*m^3^. 20s of simulation in 3D took about 3 hours.

### Fibrosis model

Fibrosis was modeled by the introduction of electrically uncoupled unexcitable nodes [29]. The local percentage of fibrosis determined the probability for a node of the computational grid to become an unexcitable obstacle, meaning that for high percentages of fibrosis, there is a high chance for a node to be unexcitable. As previous research has demonstrated that LGE-MRI enhancement correlates with regions of fibrosis identified by histological examination [30], we linearly interpolated the SI into the percentage of fibrosis for the 3D human models.

In addition, the effect of ionic remodeling in fibrotic regions was taken into account for several results of the paper [31, 32]. To describe ionic remodeling we decreased the conductance of *I*_Na_, *I*_Kr_, and *I*_Ks_ and depending on local fibrosis level as:

$$\begin{array}{ccc} G_{\text{Na}} & = & (1-1.55\frac{f}{100\%})G_{\text{Na}}^{0}, \end{array}$$ (2)

$$\begin{array}{ccc} G_{\text{Kr}} & = & (1-1.75\frac{f}{100\%})G_{\text{Kr}}^{0}, \end{array}$$ (3)

$$\begin{array}{ccc} G_{\text{Ks}} & = & (1-2\frac{f}{100\%})G_{\text{Ks}}^{0}, \end{array}$$ (4)

where *G*_*X*_ is the peak conductance of *I*_*X*_ ionic current, $G_{X}^{0}$ is the peak conductance of the current in the absence of remodeling, and *f* is the local fibrosis level in percent. These formulas yield a reduction of 62% for *I*_Na_, of 70% for *I*_Kr_, and of 80% for *I*_Ks_ if the local fibrosis *f* is 40%. These values of reduction are, therefore, in agreement with the values published in [33, 34].

**Fig 1:**
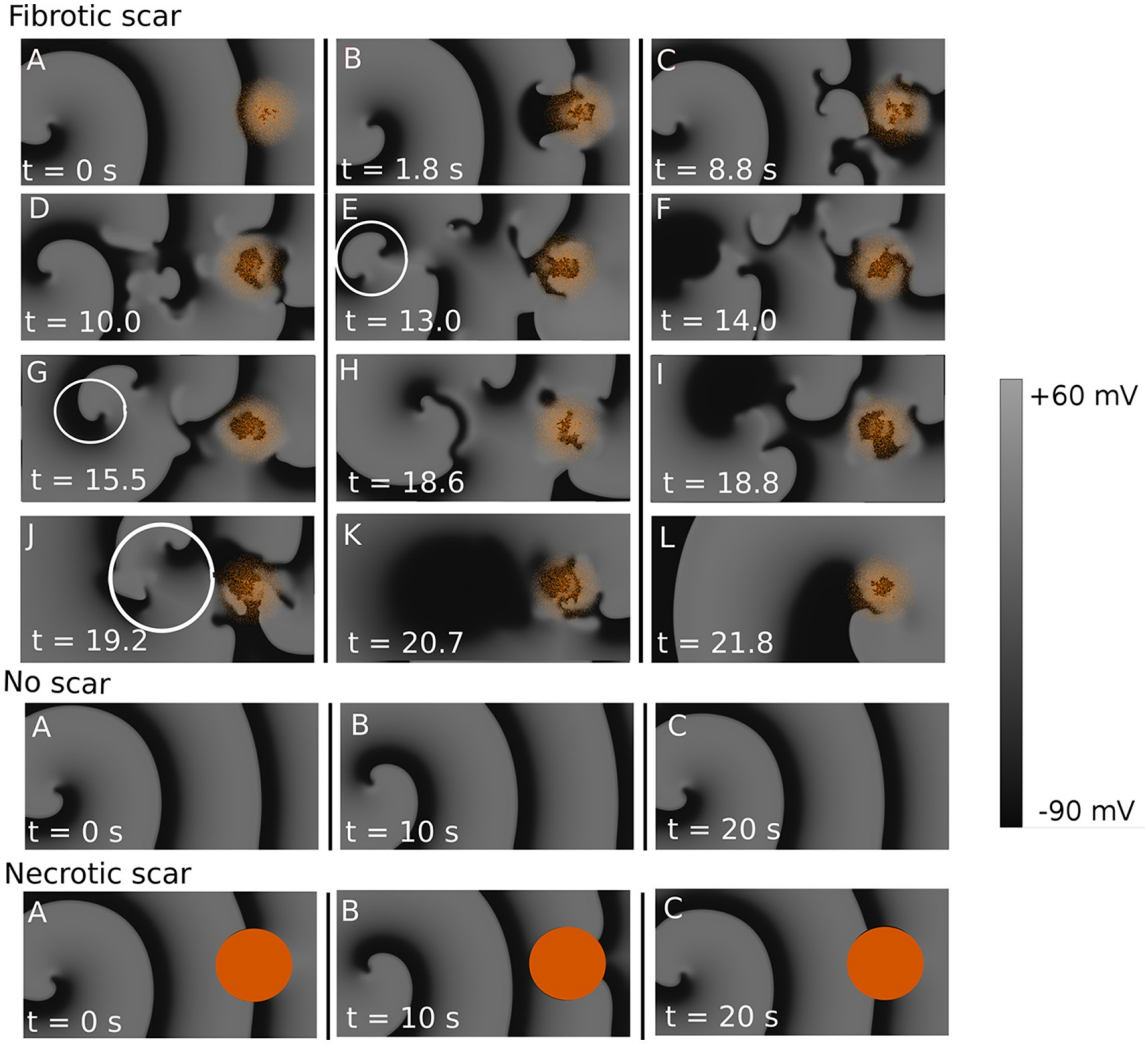
Dynamical anchoring of a distant rotor to a fibrotic region in the 2D model. Unexcitable fibrotic tissue is shown in orange and the transmembrane voltage is shown in shades of gray. Fibrotic scar: A: A rotor was initiated 15.5 cm away from the border of the fibrotic region. B: The wavefront starts to break on the scar region forming secondary sources. C-D: The secondary sources started to propagate towards the original rotor. D: The secondary sources are reaching towards the initial rotor. E: One secondary source will merge with the initial rotor, see the white circle. F: The secondary source merged with the initial rotor, in the left side of the tissue there are no rotors anymore. G: The main rotor is now indicated with the white circle, the process of rotor annihilation starts again. H-I: A new rotor annihilation results again in a dominant rotor closer to the scar. J-K: The dominant rotor merges again with a secondary source (white circle) which leads to restructuring of the activation pattern. L: The rotor is now attached to the scar. No scar A: A rotor was initiated at the left of the tissue. B-C: This rotors remains stable during the simulation of 20 s. **Necrotic scar** A: A rotor was initiated at the left of the tissue, or 15.5 cm from the border the necrotic scar. B-C: This rotors remains stable during the simulation of 20 s.

The normal conduction velocity at CL 1000 ms is 72 cm/s (CL 1000 ms). However, as the compact scar is surrounded by fibrotic tissue, the velocity of propagation in that region gradually decreases with the increase in the fibrosis percentage. For example for fibrosis of 30%, the velocity decreases to 48 cm/s (CL 1000 ms). We refer to Figure 1 in Ten Tusscher et al [25] for the planar conduction velocity as a function of the percentage fibrosis in 2D tissue and 3D tissue.

### Model of the human left ventricle

The geometry and extent of fibrosis in the human left ventricles were determined using the LGE MRI data. The normalized signal intensity was used to determine the density of local fibrosis. The fiber orientation is presented in detail in the supplementary S1 Appendix.

### Numerical methods and implementation

The model for cardiac tissue was solved by the forward Euler integration scheme with a time step of 0.02 ms. The numerical solver was implemented using the CUDA toolkit for performing the computations on graphics processing units. Simulations were performed on a GeForce GTX Titan Black graphics card using single precision calculations.

The eikonal equations for anisotropy generation were solved by the fast marching Sethian’s method [35]. The eikonal solver and the 3D model generation pipeline were implemented in the OCaml programming language.

### Rotor initiation, termination and anchoring and pseudo-ECG computation

Rotors were initiated by an S1S2 protocol, as shown in the supplementary S1 Fig. Similarly, in the whole heart simulations, spiral waves (or scroll waves) were created by an S1S2 protocol.

For the compact scar geometry used in our simulations the rotation of the spiral wave was stationary, the period of rotation of the anchored rotor was always more than 280 ms, while the period of the spiral wave was close to 220 msec. Therefore, we determined anchoring as follows: if the period of the excitation pattern was larger than 280 ms over a measuring time interval of 320 ms we classified the excitation as anchored. When the type of anchoring pattern was important (single or multi-armed spiral wave) we determined it visually. If in all points of the tissue, the voltage was below -20 mV, the pattern was classified as terminated. We applied the classification algorithm at t = 40 s in the simulation.

In the whole heart, the pseudo ECGs were calculated by assuming an infinite volume conductor and calculating the dipole source density of the membrane potential *V*_*m*_ in all voxel points of the ventricular myocardium, using the following equation [36]

$$\begin{array}{c} ECG\left(t\right)=\int \frac{\left(\vec{r},D\left(\vec{r}\right)\vec{\nabla }V\left(t\right)\right)}{|\vec{r}{|}^{3}}d^{3}r \end{array}$$ (5)

whereby *D* is the diffusion tensor, V is the voltage, and $\vec{r}$ is the vector from each point of the tissue to the recording electrode. The recording electrode was placed 10 cm from the center of the ventricles in the transverse plane.

### Clinical ECG recordings

Twelve-lead ECGs of all induced ventricular tachycardia (VT) of patients with prior myocardial infarction who underwent radiofrequency catheter ablation (RFCA) for monomorphic VT at LUMC were reviewed. All patients provided informed consent and were treated according to the clinical protocol. Programmed electrical stimulation (PES) is routinely performed before RFCA to determine inducibility of the clinical/presumed clinical VT. All the patients underwent PES and ablation according to the standard clinical protocol, therefore no ethical approval was required. Ablation typically targets the substrate for scar-related reentry VT. After ablation PES is repeated to test for re-inducibility and evaluate morphology and cycle length of remaining VTs. The significance of non-clinical, fast VTs is unclear and these VTs are often not targeted by RFCA. PES consisted of three drive cycle lengths (600, 500 and 400 ms), one to three ventricular extrastimuli (≥200 ms) and burst pacing (CL ≥200 ms) from at least two right ventricular (RV) sites and one LV site. A positive endpoint for stimulation is the induction of any sustained monomorphic VT lasting 30 s or requiring termination. ECG and intracardiac electrograms (EG) during PES were displayed and recorded simultaneously on a 48-channel acquisition system (Prucka CardioLab EP system, GE Healthcare, USA) for off-line analysis.

## Results

### Dynamical anchoring of a distant rotor in the 2D model of cardiac tissue

Fibrotic scars can not only anchor the rotors but can dynamically anchor them from a large distance. In the first experiments we studied spiral wave dynamics with and without a fibrotic scar in a generic study. The diameter of the fibrotic region was 6.4 cm, based on the similar size of the scars from patients with documented and induced VT (see the Methods section, Magnetic Resonance Imaging). The percentage of fibrosis changed linearly from 50% at the center of the scar to 0% at the scar boundary. We initiated a rotor at a distance of 15.5 cm from the scar (Fig 1, panel A) which had a period of 222 ms and studied its dynamics.

First, after several seconds the activation pattern became less regular and a few secondary wave breaks appeared at the fibrotic region (Fig 1, panel B). These irregularities started to propagate towards the tip of the initial rotor (Fig 1, panel C-D) creating a complex activation picture in between the scar and the initial rotor. Next, one of the secondary sources reached the tip of the original rotor (Fig 1, panel E). Then, this secondary source merged with the initial rotor (Fig 1, panel F), which resulted in a deceleration of the activation pattern and promoted a chain reaction of annihilation of all the secondary wavebreaks in the vicinity of the original rotor. At this moment, a secondary source located more closely to the scar dominated the simulation (Fig 1, panel G). The whole process now started again (Fig 1, panels H-K), until finally only one source became the primary source anchored to the scar (Fig 1L) with a rotation period of 307 ms. For clarity, a movie of this process is provided as supplementary S1 Movie.

**Fig 2:**
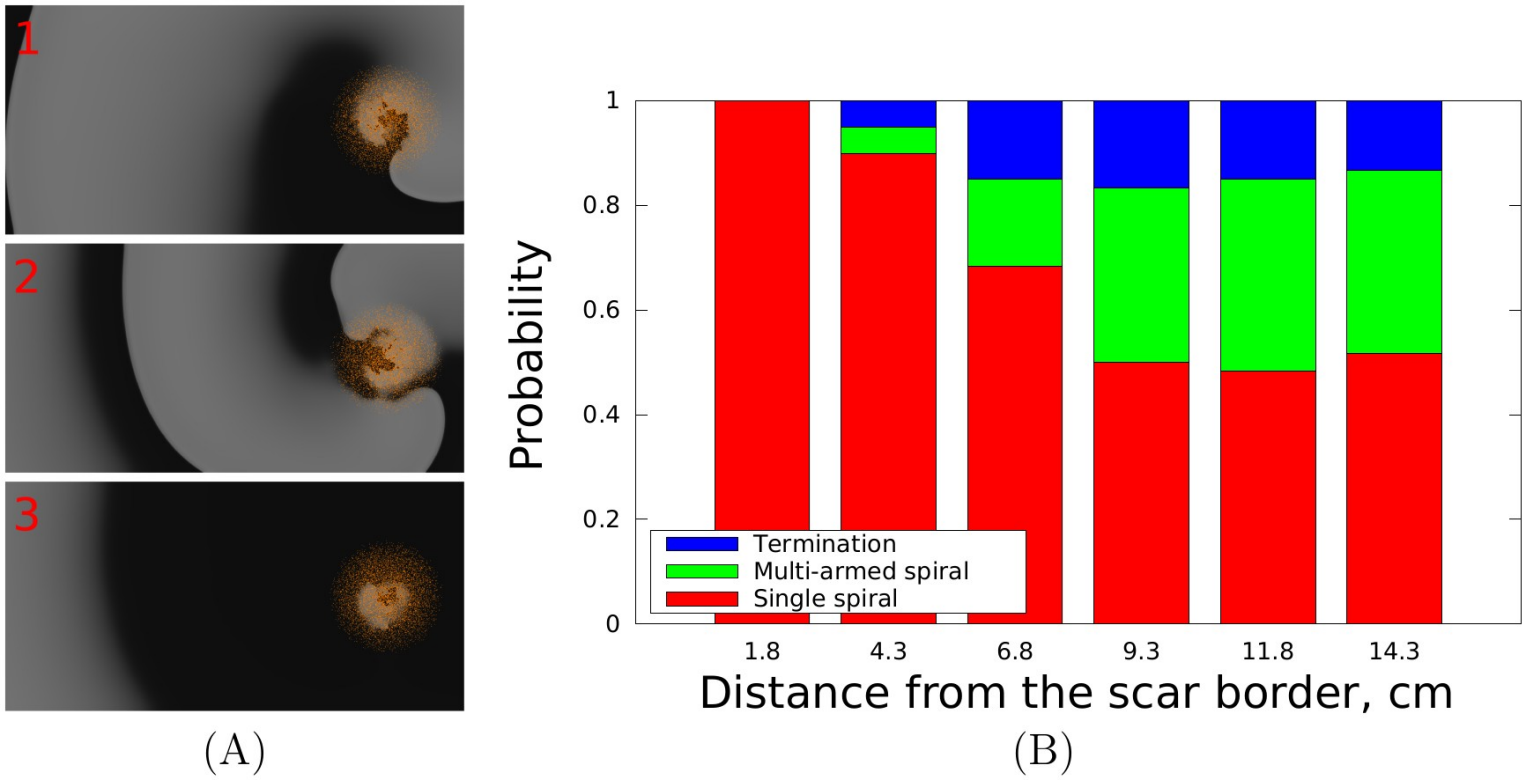
Panel A: Types of the resulting activation patterns: (1) a rotor rotating in the opposite direction than the original rotor, (2) a two-armed rotor and (3) termination of activity. Panel B: Type of the resulting activation pattern as a function of the distance between the rotor and the border of the scar. Red indicates: the resulting activation pattern is a rotor rotating either in the same direction as the original rotor or in the opposite direction. Green indicates: the resulting activation pattern is a rotor with two or three arms. Blue indicates: the electrical activity vanishes after dynamical restructuring of the activation pattern.

Note that this process occurs only if a scar with surrounding fibrotic zone was present. In the simulation entitled as ‘No scar’ in Fig 1, we show a control experiment when the same initial conditions were used in tissue without a scar. In the panel entitled as ‘Necrotic scar’ in Fig 1, a simulation with only a compact region without the surrounding fibrotic tissue is shown. In both cases the rotor was stable and located at its initial position during the whole period of simulation. The important difference here from the processes shown in Fig 1 (Fibrotic scar) is that in cases of ‘No scar’ and ‘Necrotic scar’ no new wavebreaks occur and thus we do not have a complex dynamical process of re-arrangement of the excitation patterns. We refer to this complex dynamical process leading to anchoring of a distant rotor as dynamical anchoring. Although this process contains a phase of complex behaviour, overall it is extremely robust and reproducible in a very wide range of conditions. In the second series of simulations, the initial rotor was placed at different distances from the scar border, ranging from 1.8 to 14.3 cm, to define the possible outcomes, see Fig 2. Here, in addition to a single anchored rotor shown in Fig 1H we could also obtain other final outcomes of dynamical anchoring: we obtained rotors rotating in the opposite direction (Fig 2A, top), double armed anchored rotors which had 2 wavefronts rotating around the fibrotic regions (Fig 2A, middle) or annihilation of the rotors (Fig 2A, bottom, which show shows no wave around the scar), which normally occurred as a result of annihilation of a figure-eight-reentrant pattern. To summarize, we therefore had the following possible outcomes:

- Termination of activity
- A rotor rotating either clockwise or counter-clockwise
- A two- or three-armed rotor rotating either clockwise or counter-clockwise

Fig 2, panel B presents the relative chance of the mentioned activation patterns to occur depending on the distance between the rotor and the border of the scar. We see, indeed, that for smaller initial distances the resulting activation pattern is always a single rotor rotating in the same direction. With increasing distance, other anchoring patterns are possible. If the distance was larger than about 9 cm, there is at least a 50% chance to obtain either a multi-armed rotor or termination of activity. Also note that such dynamical anchoring occurred from huge distances: we studied rotors located up to 14 cm from the scar. However, we observed that even for very large distances such as 25 cm or more such dynamical anchoring (or termination of the activation pattern) was always possible, provided enough time was given.

**Fig 3:**
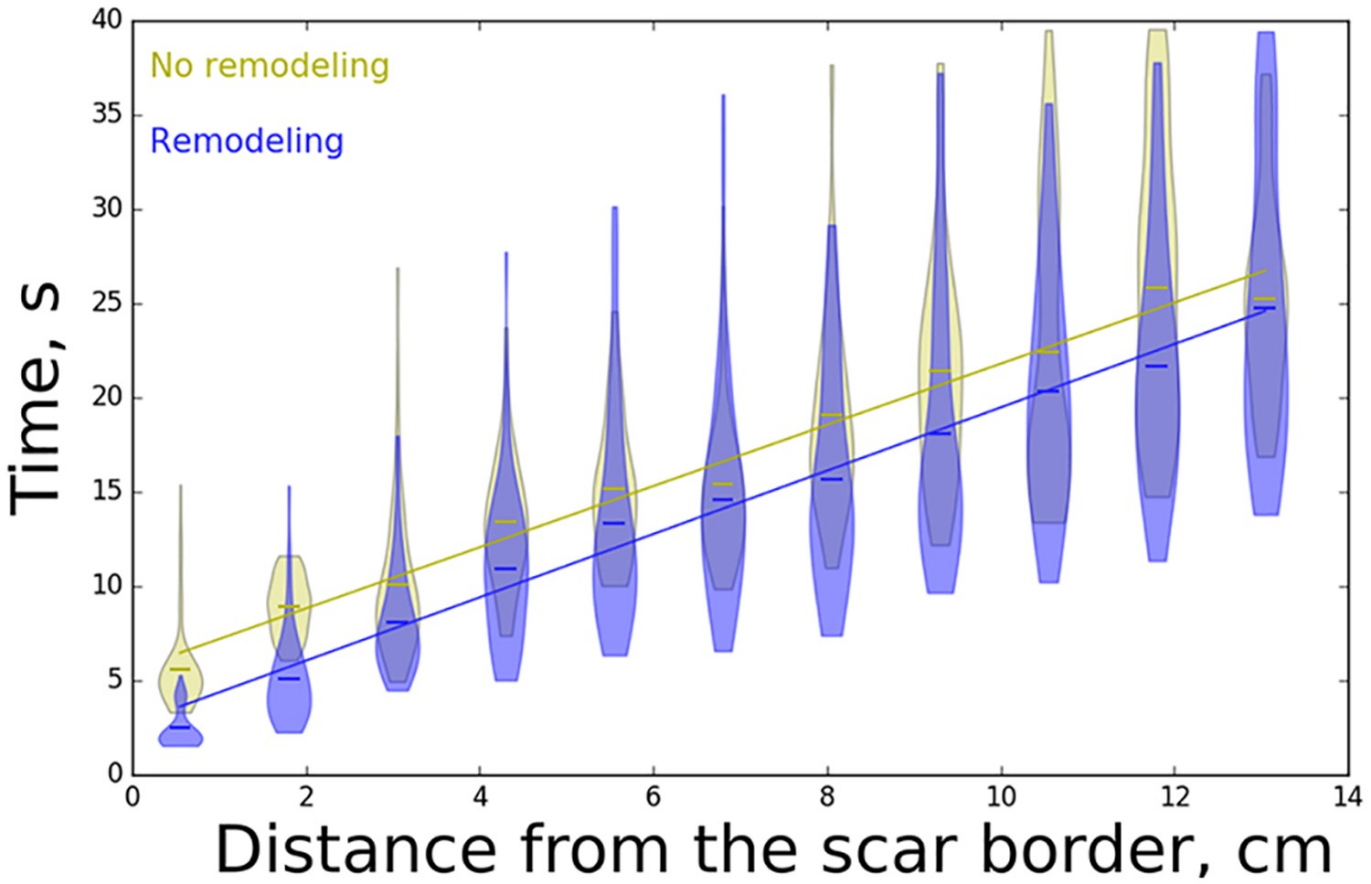
Violin plot of the dependency of time required for rotor anchoring or termination on the initial distance between the rotor tip and the border of the fibrotic region in the 2D model. Yellow indicates the model of fibrosis whereby ionic remodeling was taking into account, while blue indicates that fibrosis is modeled only by introduction of small unexcitable obstacles.

We measured the time required for the anchoring of rotors as a function of the distance from the scar. For each distance, we performed about 60 computations using different seed values of the random number generator, both with and without taking ionic remodeling into account. The results of these simulations are shown in Fig 3. We see that the time needed for dynamical anchoring depends linearly on the distance between the border of the scar and the initial rotor. The blue and yellow lines correspond to the scar model with and without ionic remodeling, respectively (ionic remodeling was modelled by decreasing the conductance of *I*_Na_, *I*_Kr_, and *I*_Ks_ as explained in the Methods Section). We interpret these results as follows; The anchoring time is mainly determined by the propagation of the chaotic regime towards the core of the original rotor and this process has a clear linear dependency. For distant rotors, propagation of this chaotic regime mainly occurs outside the region of ionic remodelling, and thus both curves in Fig 3 have the same slope. However, in the presence of ionic remodelling, the APD in the scar region is prolonged. This creates a heterogeneity and as a consequence the initial breaks in the scar region are formed about 3.5 s earlier in the scar model with remodeling compared with the scar model without remodeling.

**Fig 4:**
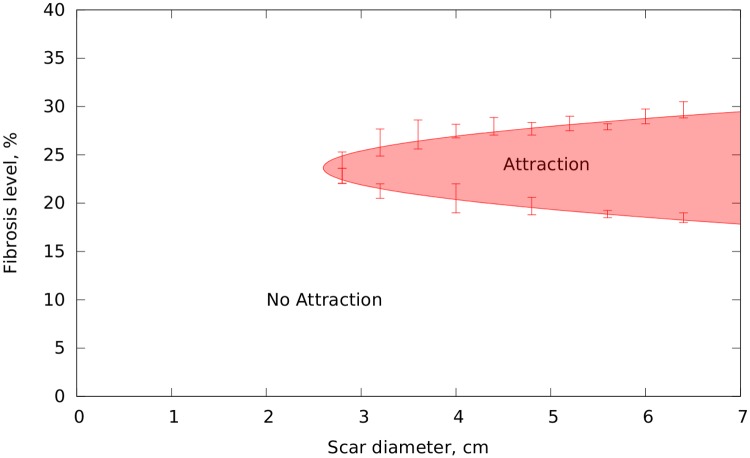
Phase diagram showing the region where the anchoring effect is present in two-parametric space: The size of the scar and the fibrosis level. The scar had a uniform fibrosis distribution. The mark “Attraction” corresponds to the region where the dynamical anchoring was obtained in more than 65% of the cases. “No Attraction” shows the region where the effect occurred in less than 65% of the cases. The scar was located 5 cm from the scar.

To identify some properties of the substrate necessary for the dynamical anchoring we varied the size and the level of fibrosis within the scar and studied if the dynamical anchoring was present. Due to the stochastic nature of the fibrosis layout we performed about 300 computations with different textures of the fibrosis for each given combination of the scar size and the fibrosis level. The results of this experiment are shown in Fig 4. Dynamical anchoring does not occur when the scar diameter was below 2.6 cm, see Fig 4. For scars of such small size we observed the absence of both the breakup and dynamical anchoring. We explain this by the fact that if the initial separation of wavebreaks formed at the scar is small, the two secondary sources merge immediately, repairing the wavefront shape and preventing formation of secondary sources [37].

Also, we see that this effect requires an intermediate level of fibrosis density. For small fibrosis levels no secondary breaks are formed (close to the boundary of the fibrotic tissue). Also, no breaks could be formed if the fibrosis level is larger than 41% in our 2D model (i.e. closer to the core), as the tissue behaves like an inexcitable scar. For a fibrosis > 41% the scar effectively becomes a large obstacle that is incapable of breaking the waves of the original rotor [37]. Close to the threshold of 41% we have also observed another interesting pattern when the breaks are formed inside the core of the scar (inside the > 41% region) only and cannot exit to the surrounding tissue, see the supplementary S1 Movie.

Finally, note that Fig 4 illustrates only a few factors important for the dynamical anchoring in a simple setup in an isotropic model of cardiac tissue. The particular values of the fibrosis level and the size of the scar can also depend on anisotropy, the texture of the fibrosis and its possible heterogeneous distribution.

### Dynamical anchoring in the patient specific model of the left ventricle

**Fig 5:**
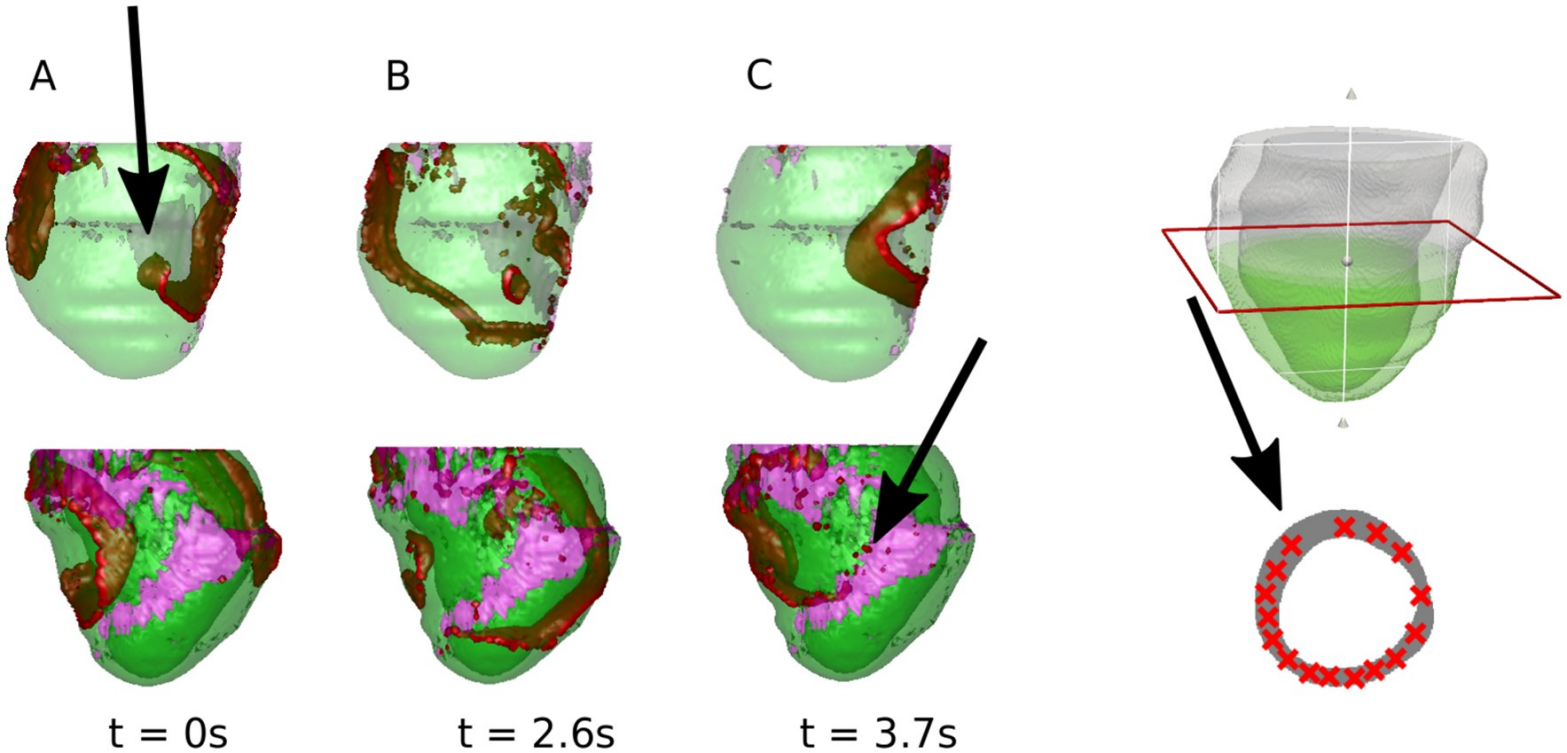
Effect of the dynamical anchoring of a rotor for a patient-specific model of the left ventricle after infero-lateral MI. Red color corresponds to high transmembrane voltage, pink color shows the scar. The top row shows the left ventricle in a modified anterior view (where the spiral was initiated) and the second row in a modified posterior view (location of scar). A: A rotor is initiated 5 cm away from the scar region. B: The breakups form making the activation pattern less regular (2.6 s after the initialization) C: The rotor gets anchored to the scar and rotates around it persistently (3.7 s after the initialization). D: The 16 different locations of the initial core of the rotor in a slice at 4 cm from the apex. In the bottom row, the simulations are shown where there is no scar present, and the spiral core stays around the same location. For a movie of the scar simulation, see the supplementary S2 Movie.

To verify that the dynamical anchoring takes place in a more realistic geometry, we developed and investigated this effect in a patient-specific model of the human left ventricle, see the Method section for details. The scar in this dataset has a complex geometry with several compact regions with size around 5-7 cm in which the percentage of fibrosis changes gradually from 0% to 41% at the core of the scar based on the imaging data, see Methods section. The remodeling of ionic channels at the whole scar region was also included to the model (including borderzone as described the Fibrosis Model in the method section). We studied the phenomenon of dynamical anchoring for 16 different locations of cores of the rotor randomly distributed in a slice of the heart at about 4 cm from the apex (see Fig 5).

Cardiac anisotropy was generated by a rule-based approach described in details in the Methods section (Model of the Human Left Ventricle). Of the 16 initial locations, shown in Fig 5, there was dynamical anchoring to the fibrotic tissue in all cases, with and without ionic remodeling. After the anchoring, in 4 cases the rotor annihilated. The effect of the attraction was augmented by the electrophysiolical remodelling, similar as in 2D.

A representative example of our 3D simulations is shown in Fig 5. We followed the same protocol as for the 2D simulations. The top 2 rows the modified anterior view and the modified posterior view in the case the scar was present. In column A, we see the original location of the spiral core (5 cm from the scar) indicated with the black arrow in anterior view. In column B, breaks are formed due to the scar tissue, and the secondary source started to appear. After 3.7 s, the spiral is anchored around the scar, indicated with the black arrow in the posterior view, and persistently rotated around it. In the bottom row, we show the same simulation but the scar was not taken into account. In this case, the spiral does not change its original location (only a slight movement, see the black arrows).

**Fig 6:**
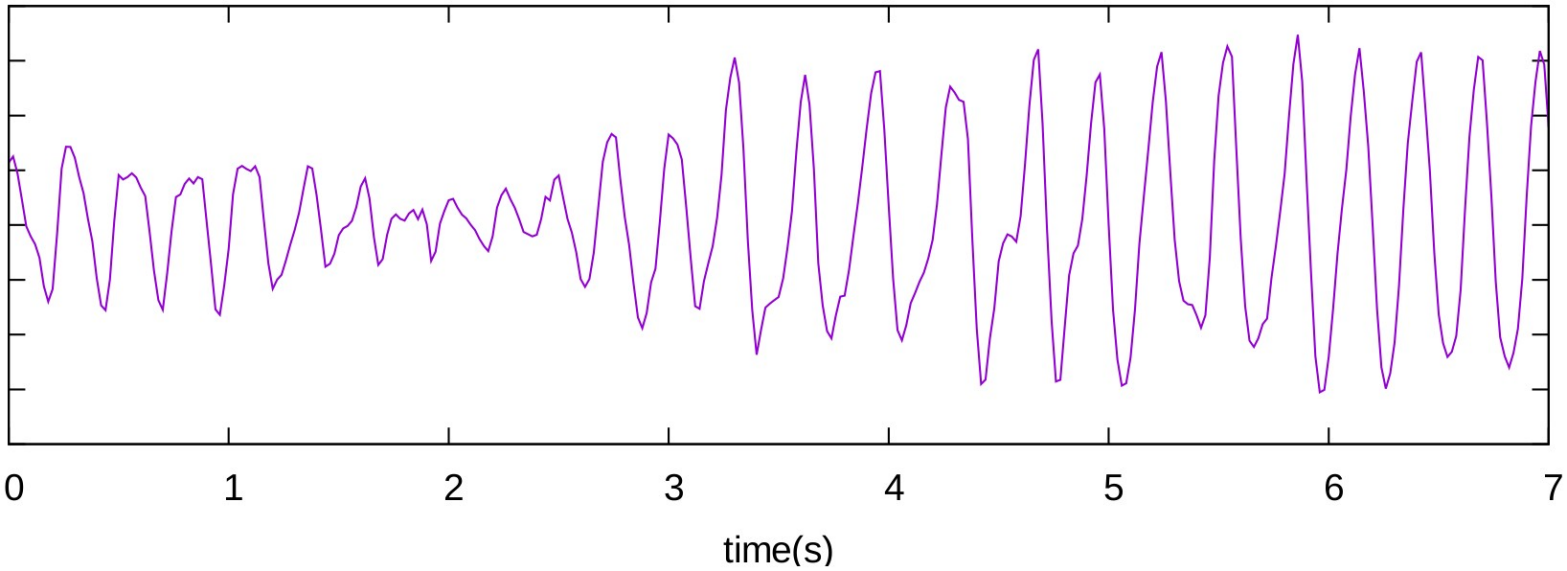
A representative example of a computed ECG for the anchoring effect in the 3D model is shown. The ECG is displayed in purple, without showing the units as only relative units are important. Anchoring is observed approximately at the end of the third second. The coupling interval increased after that time.

To evaluate if this effect can potentially be registered in clinical practice we computed the ECG for our 3D simulations. The ECG that corresponds to the example in Fig 5 is shown in Fig 6. During the first three seconds, the ECG shows QRS complexes varying in amplitude and shape and then more uniform beat-to-beat QRS morphology with a larger amplitude. This change in morphology is associated with anchoring of the rotor which occurs around three seconds after the start of the simulation. The initial irregularity is due to the presence of the secondary sources that have a slightly higher period than the original rotor. After the rotor is anchored, the pattern becomes relatively stable which corresponds to a regular saw-tooth ECG morphology. Additional ECGs for the cases of termination of the arrhythmia and anchoring are shown in supplementary S2 Fig. For the anchoring dynamics we see similar changes in the ECG morphology as in Fig 6.

The dynamical anchoring is accompanied by an increase of the cycle length (247 ± 16 ms versus 295 ± 30 ms). The reason for this effect is that the rotation of the rotor around an obstacle –anatomical reentry– is usually slower than the rotation of the rotor around its own tip—functional reentry, which is typically at the limit of cycle length permitted by the ERP.

### Clinical ECGs related to the dynamical anchoring process

**Fig 7:**
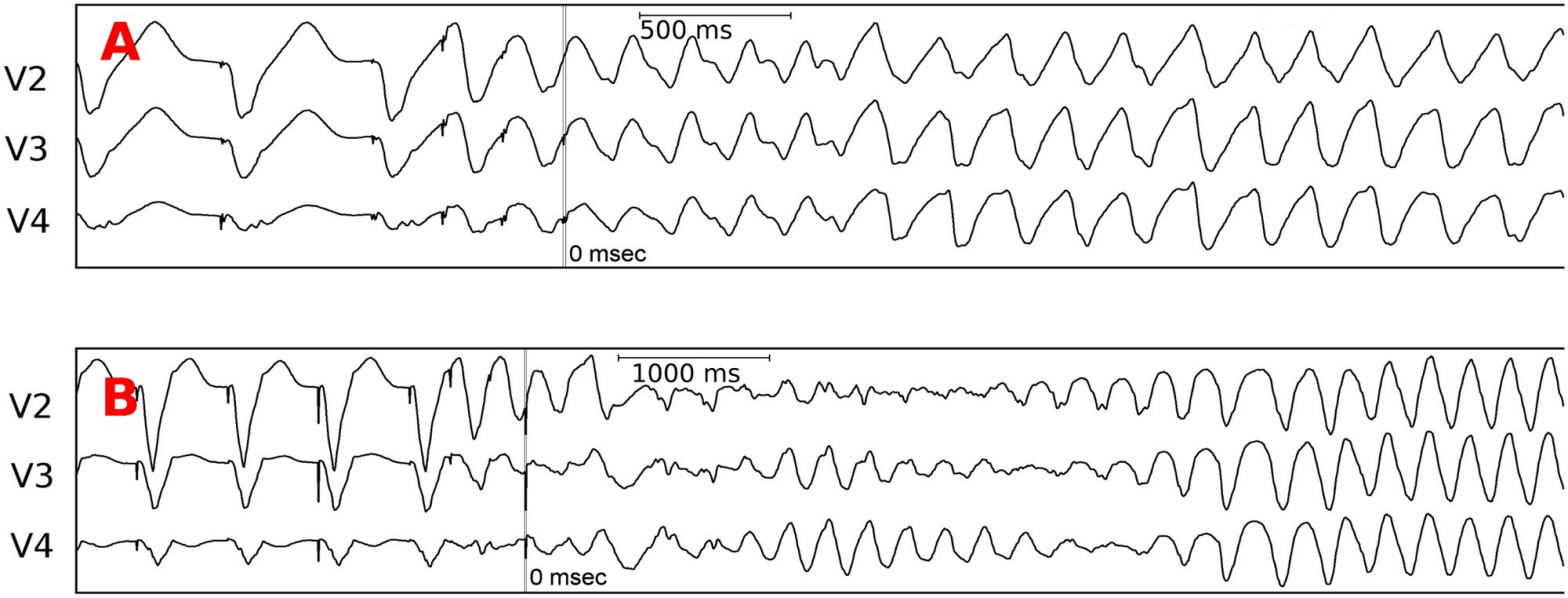
Precordial ECG leads V2, V3, and V4 recorded during induction of a ventricular tachycardia in two patients with scars in the left ventricle. First beats are paced beats; the mark “0 msec” indicates the last extrastimuli during programmed ventricular stimulation.

In the previous section, we showed that the described results on dynamical anchoring in an anatomical model of the LV of patients with post infarct scars correspond to the observations on ECGs during initiation of a ventricular arrhythmia. After initiation, in 18 out of 30 patients (60%) a time dependent change of QRS morphology was observed. Precordial ECG leads V2, V3 and V4 from two patients are depicted in Fig 7. For both patients the QRS morphology following the extra stimuli gradually changed, but the degree of changes here was different. In patient A, this morphological change is small and both parts of the ECG may be interpreted as a transition from one to another monomorhpic ventricular tachycardia (MVT) morphology. However, for patient B the transition from polymorphic ventricular tachycardia (PVT) to MVT is more apparent. In the other 16 cases we observed different variations between the 2 cases presented in Fig 7. Supplementary S3 Fig shows examples of ECGs of 4 other patients. Here, in patients 1 and 2, we see substantial variations in the QRS complexes after the arrhythmia initiation and subsequently a transformation to MVT. The recording in patient 3 is less polymorphic and in patient 4 we observe an apparent shift of the ECG from one morphology to another. It may occur, for example, if due to underlying tissue heterogeneity additional sources of excitation are formed by the initial source. Overall, the morphology with clear change from PVT to MVT was observed in 5/18 or 29% of the cases. These different degrees of variation in QRS morphology may be due to many reasons, namely the proximity of the created source of arrhythmia to the anchoring region, the underlying degree of heterogeneity and fibrosis at the place of rotor initiation, complex shape of scar, etc.

Although this finding is not a proof, it supports that the anchoring phenomenon may occur in clinical settings and serve as a possible mechanism of fast VT induced by programmed stimulation.

## Discussion

### Main results

In this study, we investigated the dynamics of arrhythmia sources –rotors– in the presence of fibrotic regions using mathematical modeling. We showed that fibrotic scars not only anchor but also induce secondary sources and dynamical competition of these sources normally results their annihilation. As a result, if one just compares the initial excitation pattern in Fig 1A and final excitation pattern in Fig 1L, it may appear as if a distant spiral wave was attracted and anchored to the scar. However, this is not the case and the anchored spiral here is a result of normal anchoring and competition of secondary sources which we call dynamical anchoring. This process is different from the usual drift or meandering of rotors where the rotor gradually changes its spatial position. In dynamical anchoring, the break formation happens in the fibrotic scar region, then it spreads to the original rotor and merges with this rotor tip and reorganizes the excitation pattern. This process repeats itself until a rotor is anchored around the fibrotic scar region. Dynamical anchoring may explain the organization from fast polymorphic to monomorphic VT, also accompanied by prolongation in CL, observed in some patients during re-induction after radio frequency catheter ablation of post-infarct scar related VT.

In our simulations the dynamics of rotors in 2D tissue were stable and for given parameter values they do not drift or meander. This type of dynamics was frequently observed in cardiac monolayers [38, 39] which can be considered as a simplified experimental model for cardiac tissue. We expect that more complex rotor dynamics would not affect our main 2D results, as drift or meandering will potentate the disappearance of the initial rotor and thus promote anchoring of the secondary wavebreaks. In our 3D simulations in an anatomical model of the heart, the dynamics of rotors is not stationary and shows the ECG of a polymorphic VT (Fig 6).

### Novel mechanism: Dynamical anchoring

The dynamical anchoring combines several processes: generation of new breaks at the scar, spread of breaks toward the original rotor, rotor disappearance and anchoring or one of the wavebreaks at the scar. The mechanisms of the formation of new wavebreaks at the scar has been studied in several papers [15, 37, 40] and can occur due to ionic heterogeneity in the scar region or due to electrotonic effects [40]. However the process of spread of breaks toward the original rotors is a new type of dynamics and the mechanism of this phenomenon remains to be studied. To some extent it is similar to the global alternans instability reported in Vandersickel et al. [41]. Indeed in Vandersickel et al. [41] it was shown that an area of 1:2 propagation block can extend itself towards the original spiral wave and is related to the restitution properties of cardiac tissue. Although in our case we do not have a clear 1:2 block, wave propagation in the presence of breaks is disturbed resulting in spatially heterogeneous change of diastolic interval which via the restitution effects can result in breakup extension. This phenomenon needs to be further studied as it may provide new ways for controlling rotor anchoring processes and therefore can affect the dynamics of a cardiac arrhythmia.

In this paper, we used the standard method of representing fibrosis by placement of electrically uncoupled unexcitable nodes with no-flux boundary conditions. Although such representation is a simplification based on the absence of detailed 3D data, it does reproduce the main physiological effects observed in fibrotic tissue, such as formation of wavebreaks, fractionated electrograms, etc [22]. The dynamical anchoring reported in this paper occurs as a result of the restructuring of the activation pattern and relies only on these basic properties of the fibrotic scar, i.e. the ability to generate wavebreaks and the ability to anchor rotors, which is reproduced by this representation. In addition, for each data point, we performed simulations with at least 60 different textures. Therefore, we expect that the effect observed in our paper is general and should exist for any possible representation of the fibrosis. The specific conditions, e.g. the size and degree of fibrosis necessary for dynamical anchoring may depend on the detailed fibrosis structure and it would be useful to perform simulations with detailed experimentally based 3D structures of the fibrotic scars, when they become available.

### Ionic heterogeneities

Similar processes can not only occur at fibrotic scars, but also at ionic heterogeneities. In Defauw et al. [42], it has been shown that rotors can be attracted by ionic heterogeneities of realistic size and shape, similar to those measured in the ventricles of the human heart [43]. These ionic heterogeneities had a prolonged APD and also caused wavebreaks, creating a similar dynamical process as described in Fig 1. In this study however, we demonstrated that structural heterogeneity is sufficient to trigger this type of dynamical anchoring.

### Different models of fibrosis

It is important to note that in this study fibrosis was modeled as regions with many small inexcitable obstacles. However, the outcome can depend on how the cellular electrophysiology and regions of fibrosis have been represented. In modeling studies, regions of fibrosis can also be represented by coupled elements with a fixed resting potential or with detailed fibroblast models, or by smoothly varying but reduced diffusion [44]. However, different in-silico studies [14, 45, 46] on modeling fibrosis in AF demonstrated that in AF, the reentrant activities co-located at the borders of fibrotic regions, although different methodologies were used to model fibrosis. For example, in a reecent modeling study by Morgan et al [45], rotors also stabilize in the border zones of patchy fibrosis in 3D atria, although fibrosis was modeled with myocyte-fibroblast coupling.

These results agree with multiple experimental and clinical studies, which also showed co-localization of rotors and fibrosis [5, 7, 8, 11–14]. Our results suggest that the possible mechanisms for the fact that such patterns are so abundant is due to dynamical anchoring. Therefore, we expect that different methodologies in modeling studies would give rise to similar results.

### Stability and final location of the rotor

In our simulations the dynamics of rotors in 2D tissue were stable and for given parameter values they do not drift or meander. Although this type of dynamics was observed in cardiac monolayers [39, 47], the size of the myocardial cell in cultured monolayer is usually smaller than myocytes in myocardium, and they do not have a cylindrical form. Moreover, the gap junctions in cell cultures are usually found circumferentially, whereas in vivo gap junctions are found mostly at intercalated disks [48]. As these differences may affect the stabilty of the rotors, i.e. cause more complex dynamics, and it may also affect the results of 2D studies.

Another possible effect by which fibrotic scars can influence rotor dynamics is the electrotonic influence from the fibrotic scar region. Indeed, electrotonic effects are well known in cardiac electrophysiology and can strongly affect the heterogeneity of cardiac tissue and the susceptibility to arrhythmias [49]. It was also estimated that, in many models of cardiac cells, the spatial length of the electrotonic effects is of the order of 0.5-1cm [50, 51]. In our case we see dynamical anchoring for spirals located as far as 10-12 cm, which is far beyond these values. In addition, we also observe the same effect in case if we do not have ionic remodelling in the scar region (Fig 3), and thus in that case AP of all cells are the same (up to some possible boundary effects). Therefore, we think that the electrotonic influence from the scar is unlikely to be a main determinant of the dynamic anchoring. However, a heterogeneity around the scar has some effect on the anchoring process which can also be seen in Fig 3.

Although the dynamical anchoring reported in this paper will always bring the rotor to the scar region, the precise location of the rotor inside this region was not studied here. This question requires additional investigation, which is currently being performed in our research group. The first preliminary results indicate that in case of a scar with a complex structure multiple anchoring sites are possible. Their location depends on several factors, such as the location of the initial rotor, the presence and the extent of the ionic remodelling. In this work, to describe ionic remodeling we decreased the conductance of *I*_Na_, *I*_Kr_, and *I*_Ks_ as explained in the Methods Section. Identification of the specific features of the anchoring region and its delineation is of great importance as it may have implications for the treatment of the related arrhythmias. It would also be of value to quantify the excitation patterns in terms of number of re-entrant sources during the dynamic anchoring process, which can be done using the methodology in Panfilov et al [52] and or Vandersickel et al [53].

### Slow conductive channels

The most common mechanism of scar-related VT is due to slow conduction through a surviving channel in the scar. In this manuscript we did not model it explicitly. This is because we wanted to investigate a general phenomenon which can occur in cardiac tissue in the presence of fibrosis and did not try to reproduce specific geometries of the scar and slow conducting channels. It would be interesting to perform similar studies based on detailed reconstructions of infarction scars, such as [54] and see if the presence of the slow conduction channel(s) would affect the process of anchoring. However, note that complex patterns of fibrosis similar to those studied in our paper were observed for example in patients with non-ischaemic cardiomyopathy [55]. This type of substrate can also result in monomorphic ventricular tachycardia typical for anchored rotational activity.

### Limitations

In this paper, we considered the case of a pre-existing rotor and focused on its interaction with the fibrotic scar. The formation of a rotor can occur via multiple possible mechanisms (e.g. [15–18]) which we did not take into account. This is because we wanted to address the process at a stage common for all mechanisms. This assumption is idealized, and it would be more natural to consider the complete sequence of transition from the sinus rhythm to rotor formation and then to its interaction with the scar. This is because such interaction process can potentially influence the process of dynamic anchoring. However, such interactions will add additional levels of complexity to the problem on top of the effects studied in our paper and may be specific for each particular mechanism. Therefore we decided to focus on this later stage of an already existing rotor, which is common for all these mechanisms. This is a limitation of our approach and it should be addressed in subsequent studies for each of the mechanisms of generation of the initial rotor.

We have studied the dependency of dynamical anchoring to the scar on size and fibrotic content of the scar in a simplified situation: the scar was of circular shape, the fibrosis was modelled as diffuse fibrosis and had a constant level within the entire scar (Fig 4). It would be of value to extend such studies and consider different shapes of the scar and study the possible effects of this shape on the anchoring. Additionally, we could consider a non-homogeneous distribution of fibrosis inside the scar and study it with and without of ionic heterogeneity. Furthermore, it would be valuable to study the possible effects of different texture of fibrosis like interstitial and patchy patterns on the dynamical anchoring. Also, in this paper we have always considered an initial rotor rotating in a homogeneous part of the tissue. It would be important to study more realistic tissue setups, where fibrosis is not only present around the scar but also at distant locations where the rotor is present. We plan to address these shortcomings in subsequent research. Our 2D simulations are performed for rotors which have stationary rotation. As stationary rotation of free non-anchored rotors is unlikely to occur in the whole heart, these results account for an idealized situation and may change if the rotor rotation is not stationary. However, as we discussed only a qualitative relation between the data we think that our general interpretation is acceptable. Clinical ECG recordings used in our paper were taken after ablation. It would be important to compare pre-ablation ECGs showing arrhythmia dynamics in patients with collected LGE MRI images. Unfortunately such clinical information was not available to us.

Most of our simulations were performed with a space step of 250 microns. Although such step is widely used in computational studies on cardiac propagation it is larger than the typical size of cardiac cells. Therefore, it would be of value to perform simulations with a smaller space step (e.g. 100 microns). However, as 2D studies performed here use a generic representation of cardiac fibrosis we do not expect qualitative changes of the results obtained in this paper.

## Supporting information

S1 Fig Creation of a spiral in 2D with the S1S2 protocol. A plain wave (S1 stimulus) is initiated (a, b). When the wave has passed, an S2 stimulus (c) is given, which created a spiral wave).
(TIFF)

S2 Fig Additional examples of computed ECGs in the 3D model. **In the upper pannel the spiral disappeared after anchoring**. The other two cases show ECGs for the dynamics similar to that of Fig 6.
(TIFF)

S3 Fig Additional examples of precordial ECG leads V2, V2 and V3 for 4 different patients, as shown in figure, recorded during induction of a ventricular tachycardia. In patients 1 and 2, after the initiation of the arrhythmia, substantial variations in the QRS complexes are observed followed by a transformation to a MVT. The recording in patient 3 is less polymorphic and in patient 4 we observe an apparent shift of the ECG from one morphology to another.
(TIFF)

S1 Movie A video of the simulation shown in Fig 1(A)–1(L) of the dynamical anchoring of a spiral wave by a fibrotic scar. (MP4)

S2 Movie A video of the simulation shown in Fig 5. The left panel shows a modified anterior view, while the right panel shows a modified posterior view.
(MP4)

S1 Appendix Fiber orientation of the model of the left ventricle. (PDF)

## References

[1] Narayan SM, Krummen DE, Shivkumar K, Clopton P, Rappel WJ, Miller JM. Treatment of atrial fibrillation by the ablation of localized sources: CONFIRM (Conventional Ablation for Atrial Fibrillation With or Without Focal Impulse and Rotor Modulation) trial. Journal of the American College of Cardiology. 2012;60(7):628–636. 10.1016/j.jacc.2012.05.022 22818076 PMC3416917

[2] Nash MP, Mourad A, Clayton RH, Sutton PM, Bradley CP, Hayward M, et al Evidence for multiple mechanisms in human ventricular fibrillation. Circulation. 2006;114(6):536–542. 10.1161/CIRCULATIONAHA.105.602870 16880326

[3] Bradley CP, Clayton RH, Nash MP, Mourad A, Hayward M, Paterson DJ, et al Human ventricular fibrillation during global ischemia and reperfusion: paradoxical changes in activation rate and wavefront complexity. Circ Arrhythm Electrophysiol. 2011;4(5):684–691. 10.1161/CIRCEP.110.961284 21841193

[4] Krummen DE, Hayase J, Morris DJ, Ho J, Smetak MR, Clopton P, et al Rotor stability separates sustained ventricular fibrillation from self-terminating episodes in humans. J Am Coll Cardiol. 2014;63(24):2712–2721. 10.1016/j.jacc.2014.03.037 24794115 PMC4396824

[5] Nair K, Umapathy K, Farid T, Masse S, Mueller E, Sivanandan RV, et al Intramural activation during early human ventricular fibrillation. Circ Arrhythm Electrophysiol. 2011;4(5):692–703. 10.1161/CIRCEP.110.961037 21750274

[6] Jeyaratnam J, Umapathy K, Masse S, Nair K, Farid T, Krishnan S, et al Relating spatial heterogeneities to rotor formation in studying human ventricular fibrillation. Conf Proc IEEE Eng Med Biol Soc. 2011;2011:231–234. 10.1109/IEMBS.2011.6090043 22254292

[7] Ringenberg J, Deo M, Filgueiras-Rama D, Pizarro G, Ibañez B, Peinado R, et al Effects of fibrosis morphology on reentrant ventricular tachycardia inducibility and simulation fidelity in patient-derived models. Clinical Medicine Insights: Cardiology. 2014;8:CMC–S15712. 10.4137/CMC.S15712 PMC4210189 25368538

[8] Haissaguerre M, Shah AJ, Cochet H, Hocini M, Dubois R, Efimov I, et al Intermittent drivers anchoring to structural heterogeneities as a major pathophysiologic mechanism of human persistent atrial fibrillation. J Physiol. 2016 10.1113/JP270617 26890861 PMC4850206

[9] Davidenko JM, Pertsov AV, Salomonsz R, Baxter W, Jalife J. Stationary and drifting spiral waves of excitation in isolated cardiac muscle. Nature. 1992;355(6358):349–351. 10.1038/355349a0 1731248

[10] Yamazaki M, Mironov S, Taravant C, Brec J, Vaquero LM, Bandaru K, et al Heterogeneous atrial wall thickness and stretch promote scroll waves anchoring during atrial fibrillation. Cardiovasc Res. 2012;94(1):48–57. 10.1093/cvr/cvr357 22227155 PMC3307378

[11] Hansen BJ, Zhao J, Csepe TA, Moore BT, Li N, Jayne LA, et al Atrial fibrillation driven by micro-anatomic intramural re-entry revealed by simultaneous sub-epicardial and sub-endocardial optical mapping in explanted human hearts. Eur Heart J. 2015;36(35):2390–2401. 10.1093/eurheartj/ehv233 26059724 PMC4568403

[12] Skanes AC, Mandapati R, Berenfeld O, Davidenko JM, Jalife J. Spatiotemporal periodicity during atrial fibrillation in the isolated sheep heart. Circulation. 1998;98(12):1236–1248. 10.1161/01.CIR.98.12.1236 9743516

[13] Gonzales MJ, Vincent KP, Rappel WJ, Narayan SM, McCulloch AD. Structural contributions to fibrillatory rotors in a patient-derived computational model of the atria. Europace. 2014;16 Suppl 4:iv3–iv10. 10.1093/europace/euu251 25362167 PMC4565557

[14] Zahid S, Cochet H, Boyle PM, Schwarz EL, Whyte KN, Vigmond EJ, et al Patient-derived models link reentrant driver localization in atrial fibrillation to fibrosis spatial pattern. Cardiovasc Res. 2016 10.1093/cvr/cvw073 27056895 PMC4872878

[15] Arevalo H, Plank G, Helm P, Halperin H, Trayanova N. Tachycardia in post-infarction hearts: insights from 3D image-based ventricular models. PloS one. 2013;8(7):e68872 10.1371/journal.pone.0068872 23844245 PMC3699514

[16] Antzelevitch C, Burashnikov A. Overview of basic mechanisms of cardiac arrhythmia. Cardiac electrophysiology clinics. 2011;3(1):23–45. 10.1016/j.ccep.2010.10.012 21892379 PMC3164530

[17] Weiss JN, Qu Z, Chen PS, Lin SF, Karagueuzian HS, Hayashi H, et al The dynamics of cardiac fibrillation. Circulation. 2005;112(8):1232–1240. 10.1161/CIRCULATIONAHA.104.529545 16116073

[18] Rosenshtraukh LV, Zaitsev AV, Fast VG, Pertsov AM, Krinsky VI. Vagally induced block and delayed conduction as a mechanism for circus movement tachycardia in frog atria. Circulation research. 1989;64(2):213–226. 10.1161/01.RES.64.2.213 2783563

[19] Liu L, Nattel S. Differing sympathetic and vagal effects on atrial fibrillation in dogs: role of refractoriness heterogeneity. American Journal of Physiology-Heart and Circulatory Physiology. 1997;273(2):H805–H816. 10.1152/ajpheart.1997.273.2.H805 9277498

[20] Piers SRD, Tao Q, de Riva Silva M, Siebelink HM, Schalij MJ, van der Geest RJ, et al CMR-based identification of critical isthmus sites of ischemic and nonischemic ventricular tachycardia. JACC Cardiovasc Imaging. 2014;7(8):774–784. 10.1016/j.jcmg.2014.03.013 25051947

[21] Roes SD, Borleffs CJW, van der Geest RJ, Westenberg JJM, Marsan NA, Kaandorp TAM, et al Infarct tissue heterogeneity assessed with contrast-enhanced MRI predicts spontaneous ventricular arrhythmia in patients with ischemic cardiomyopathy and implantable cardioverter-defibrillator. Circ Cardiovasc Imaging. 2009;2(3):183–190. 10.1161/CIRCIMAGING.108.826529 19808591

[22] Vigmond E, Pashaei A, Amraoui S, Cochet H, Hassaguerre M. Percolation as a mechanism to explain atrial fractionated electrograms and reentry in a fibrosis model based on imaging data. Heart Rhythm. 2016 10.1016/j.hrthm.2016.03.019 26976038

[23] Mewton N, Liu CY, Croisille P, Bluemke D, Lima JAC. Assessment of myocardial fibrosis with cardiovascular magnetic resonance. Journal of the American College of Cardiology. 2011;57:891–903. 10.1016/j.jacc.2010.11.013 21329834 PMC3081658

[24] Panfilov A, Holden A Computer simulation of re-entry sources in myocardium in two and three dimensions. Journal of Theoretical Biology. 1993; 161: 271–285 10.1006/jtbi.1993.1055 8331954

[25] Ten Tusscher KH, Panfilov AV. Influence of diffuse fibrosis on wave propagation in human ventricular tissue. Europace. 2007;9(suppl_6):vi38–vi45. 10.1093/europace/eum206 17959692

[26] Kazbanov IV, ten Tusscher KHWJ, Panfilov AV. Effects of Heterogeneous Diffuse Fibrosis on Arrhythmia Dynamics and Mechanism. Scientific Reports. 2016;6:20835 10.1038/srep20835 26861111 PMC4748409

[27] ten Tusscher KH, Noble D, Noble PJ, Panfilov AV. A model for human ventricular tissue. Am J Physiol Heart Circ Physiol. 2004;286(4):H1573–1589. 10.1152/ajpheart.00794.2003 14656705

[28] ten Tusscher KH, Panfilov AV. Alternans and spiral breakup in a human ventricular tissue model. Am J Physiol Heart Circ Physiol. 2006;291(3):H1088–1100. 10.1152/ajpheart.00109.2006 16565318

[29] Ten Tusscher KH, Panfilov AV. Influence of diffuse fibrosis on wave propagation in human ventricular tissue. Europace. 2007; 9(suppl_6):vi38–45. 10.1093/europace/eum206 17959692

[30] McGann C, Akoum N, Patel A, Kholmovski E, Revelo P, Damal K, et al Atrial fibrillation ablation outcome is predicted by left atrial remodeling on MRI. Circulation: Arrhythmia and Electrophysiology. 2013; p. CIRCEP–113. 10.1161/CIRCEP.113.000689 PMC4086672 24363354

[31] Pinto JM, Boyden PA. Electrical remodeling in ischemia and infarction. Cardiovasc Res. 1999;42(2):284–297. 10.1016/S0008-6363(99)00013-9 10533567

[32] Nattel S, Maguy A, Le Bouter S, Yeh YH. Arrhythmogenic ion-channel remodeling in the heart: heart failure, myocardial infarction, and atrial fibrillation. Physiol Rev. 2007;87(2):425–456. 10.1152/physrev.00014.2006 17429037

[33] Pu J, Boyden PA. Alterations of Na+ currents in myocytes from epicardial border zone of the infarcted heart. A possible ionic mechanism for reduced excitability and postrepolarization refractoriness. Circ Res. 1997;81(1):110–119. 10.1161/01.RES.81.1.110 9201034

[34] Jiang M, Cabo C, Yao J, Boyden PA, Tseng G. Delayed rectifier K currents have reduced amplitudes and altered kinetics in myocytes from infarcted canine ventricle. Cardiovasc Res. 2000;48(1):34–43. 10.1016/S0008-6363(00)00159-0 11033106

[35] Sethian JA. A fast marching level set method for monotonically advancing fronts. Proceedings of the National Academy of Sciences. 1996;93(4):1591–1595. 10.1073/pnas.93.4.1591 PMC39986 11607632

[36] Plonsey R. The use of a bidomain model for the study of excitable media. Lect Math Life Sci. 1989;21:123–149.

[37] Majumder R, Pandit R, Panfilov AV. Turbulent electrical activity at sharp-edged inexcitable obstacles in a model for human cardiac tissue. Am J Physiol Heart Circ Physiol. 2014;307(7):H1024–H1035. 10.1152/ajpheart.00593.2013 25108011

[38] Zlochiver S, Muñoz V, Vikstrom KL, Taffet SM, Berenfeld O, Jalife J. Electrotonic myofibroblast-to-myocyte coupling increases propensity to reentrant arrhythmias in two-dimensional cardiac monolayers. Biophysical journal. 2008;95:4469–4480. 10.1529/biophysj.108.136473 18658226 PMC2567962

[39] Bingen BO, Askar SF, Schalij MJ, Kazbanov IV, Ypey DL, Panfilov AV, et al Prolongation of minimal action potential duration in sustained fibrillation decreases complexity by transient destabilization. Cardiovascular research. 2012;97(1):161–170. 10.1093/cvr/cvs288 22977009

[40] Cabo C, Pertsov AM, Davidenko JM, Baxter WT, Gray RA, Jalife J. Vortex shedding as a precursor of turbulent electrical activity in cardiac muscle. Biophysical journal. 1996;70(3):1105–1111. 10.1016/S0006-3495(96)79691-1 8785270 PMC1225040

[41] Vandersickel N, Defauw A, Dawyndt P, Panfilov AV. Global alternans instability and its effect on non-linear wave propagation: dynamical Wenckebach block and self terminating spiral waves. Scientific reports. 2016;6:29397 10.1038/srep29397 27384223 PMC4935945

[42] Defauw A, Vandersickel N, Dawyndt P, Panfilov AV. Small size ionic heterogeneities in the human heart can attract rotors. Am J Physiol Heart Circ Physiol. 2014 10.1152/ajpheart.00410.2014 25217650

[43] Glukhov AV, Fedorov VV, Lou Q, Ravikumar VK, Kalish PW, Schuessler RB, et al Transmural dispersion of repolarization in failing and nonfailing human ventricle. Circ Res. 2010;106(5):981–991. 10.1161/CIRCRESAHA.109.204891 20093630 PMC2842469

[44] Clayton RH Dispersion of recovery and vulnerability to re-entry in a model of human atrial tissue with simulated diffuse and focal patterns of fibrosis. Frontiers in physiology. 2018; 9 10.3389/fphys.2018.01052 30131713 PMC6090998

[45] Morgan R, Colman MA, Chubb H, Seemann G, Aslanidi OV Slow conduction in the border zones of patchy fibrosis stabilizes the drivers for atrial fibrillation: Insights from multi-scale human atrial modeling. Frontiers in physiology 2016 7: 474 10.3389/fphys.2016.00474 27826248 PMC5079097

[46] McDowell KS, Zahid S, Vadakkumpadan F, Blauer J, MacLeod RS, Trayanova NA. Virtual electrophysiological study of atrial fibrillation in fibrotic remodeling. PLoS One. 2015;10(2):e0117110 10.1371/journal.pone.0117110 25692857 PMC4333565

[47] Zlochiver S, Munoz V, Vikstrom KL, Taffet SM, Berenfeld O, Jalife J. Electrotonic myofibroblast-to-myocyte coupling increases propensity to reentrant arrhythmias in two-dimensional cardiac monolayers. Biophysical journal. 2008;95(9):4469–4480. 10.1529/biophysj.108.136473 18658226 PMC2567962

[48] Angst BD, Khan LU, Severs NJ, Whitely K, Rothery S, Thompson RP, et al Dissociated spatial patterning of gap junctions and cell adhesion junctions during postnatal differentiation of ventricular myocardium. Circulation research. 1997;80(1):88–94. 10.1161/01.RES.80.1.88 8978327

[49] Billman GE, del Rio CL. Cardiac electronic remodeling and susceptibility to arrhythmias: an introduction and brief historical overview. Frontiers in physiology. 2015;6:196 10.3389/fphys.2015.00196 26217235 PMC4491600

[50] Sampson KJ, Henriquez CS. Electrotonic influences on action potential duration dispersion in small hearts: a simulation study. American Journal of Physiology-Heart and Circulatory Physiology. 2005;289(1):H350–H360. 10.1152/ajpheart.00507.2004 15734889

[51] Defauw A, Kazbanov IV, Dierckx H, Dawyndt P, Panfilov AV. Action potential duration heterogeneity of cardiac tissue can be evaluated from cell properties using Gaussian Green’s function approach. PLoS one. 2013;8(11):e79607 10.1371/journal.pone.0079607 24260262 PMC3832584

[52] Panfilov A. Three-dimensional organization of electrical turbulence in the heart. Physical Review E. 1999;59(6):R6251 10.1103/PhysRevE.59.R6251 11969731

[53] Vandersickel N, Bossu A, De Neve J, Dunnink A, Meijborg VM, van der Heyden MA, et al Short-Lasting Episodes of Torsade de Pointes in the Chronic Atrioventricular Block Dog Model Have a Focal Mechanism, While Longer-Lasting Episodes Are Maintained by Re-Entry. JACC: Clinical Electrophysiology. 2017;3(13):1565–1576. 10.1016/j.jacep.2017.06.016 29759839

[54] López-Yunta M, León DG, Alfonso-Almazán JM, Marina-Breysse M, Quintanilla JG, Sánchez-González J, et al Implications of bipolar voltage mapping and magnetic resonance imaging resolution in biventricular scar characterization after myocardial infarction. EP Europace. 2018. 10.1093/europace/euy192 PMC6321957 30239689

[55] Glashan CA, Androulakis AF, Tao Q, Glashan RN, Wisse LJ, Ebert M, et al Whole human heart histology to validate electroanatomical voltage mapping in patients with non-ischaemic cardiomyopathy and ventricular tachycardia. European heart journal. 2018 10.1093/eurheartj/ehy168 29617764

