# Supplementary figures and images for: Dynamical anchoring of distant arrhythmia sources by fibrotic regions via restructuring of the activation pattern
Source: PLoS Comput Biol. 2018 Dec 20;14(12):e1006637. doi: 10.1371/journal.pcbi.1006637 (PMC6319787; doi:10.1371/journal.pcbi.1006637)

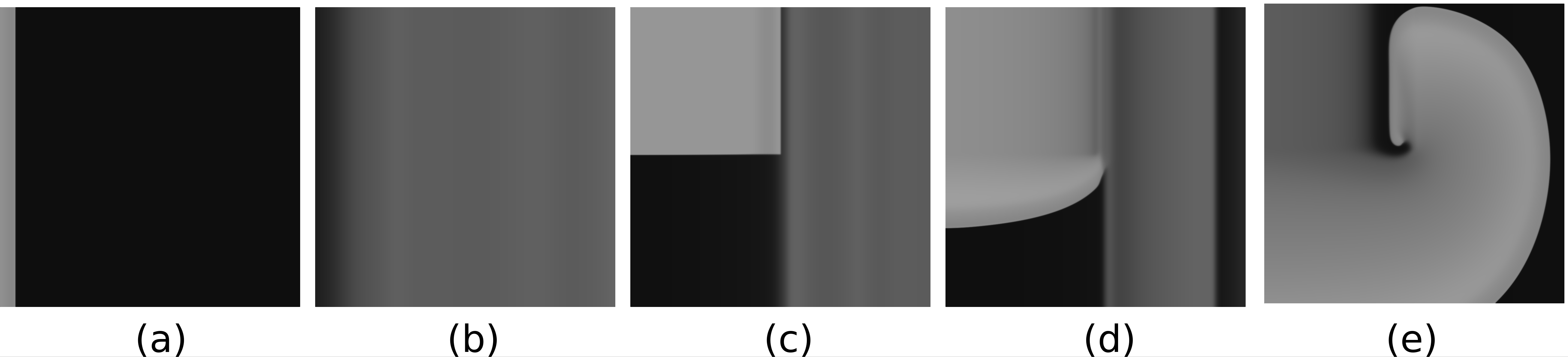

Supplement: S1 Fig — A plain wave (S1 stimulus) is initiated (a, b). When the wave has passed, an S2 stimulus (c) is given, which created a spiral wave). (TIFF) [file pcbi.1006637.s001.tiff]

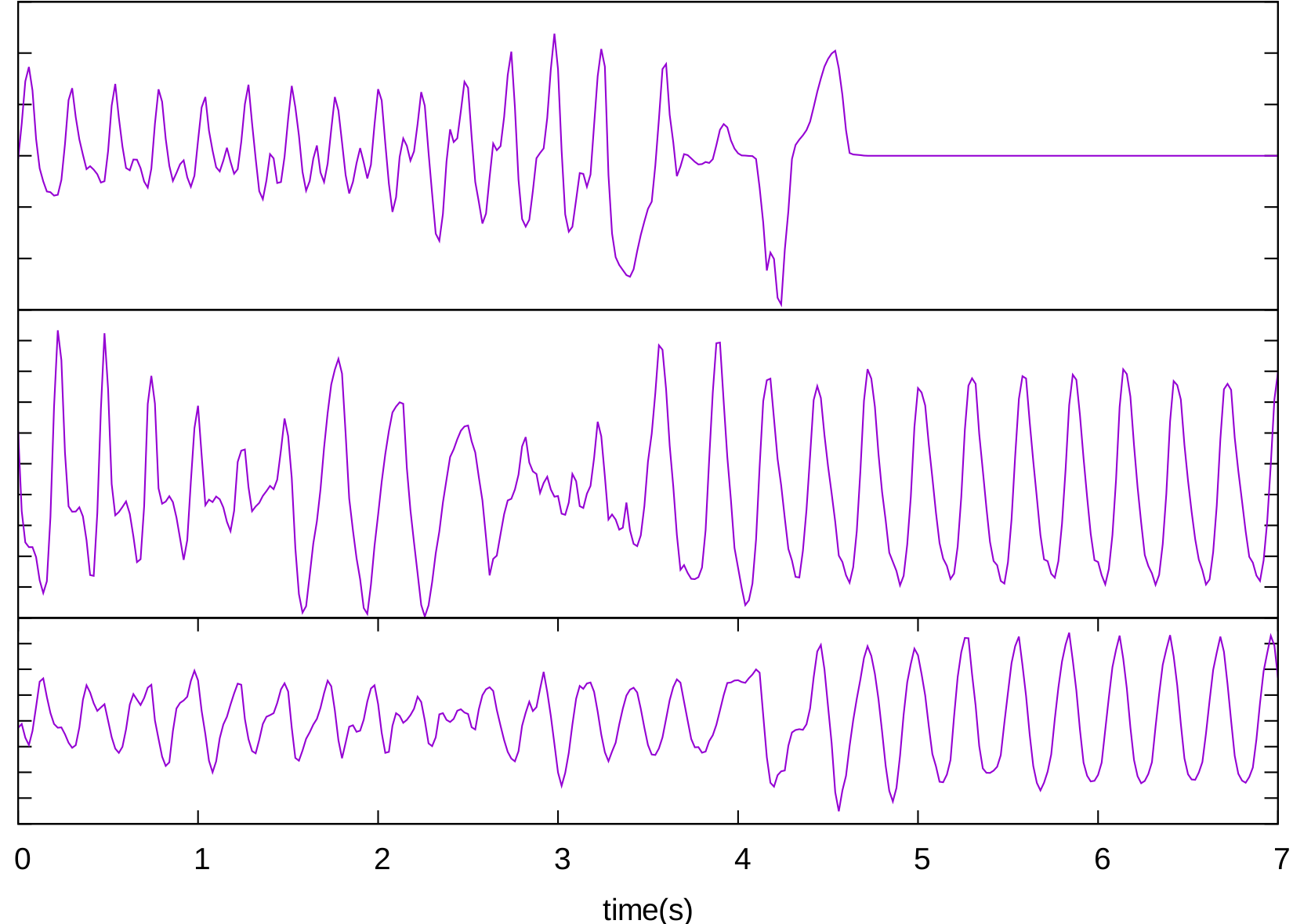

Supplement: S2 Fig — In the upper pannel the spiral disappeared after anchoring. The other two cases show ECGs for the dynamics similar to that of Fig 6. (TIFF) [file pcbi.1006637.s002.tiff]
