# Supplementary material for: Dynamical anchoring of distant arrhythmia sources by fibrotic regions via restructuring of the activation pattern
Source: PLoS Comput Biol. 2018 Dec 20;14(12):e1006637. doi: 10.1371/journal.pcbi.1006637 (PMC6319787; doi:10.1371/journal.pcbi.1006637)
Supplement: S1 Appendix — (PDF) [file pcbi.1006637.s006.pdf]

Dynamical anchoring of distant Arrhythmia Sources by Fibrotic Regions via Restructuring of the Activation Pattern

Fiber orientation of the model of the left ventricle

The anisotropy due to fiber orientation was defined using a rule-based approach based on [2], which is similar to approaches used in [3, 4] but uses a direct way for determining the distance though the myocardial wall. The fiber helix angle α_1 was taken to be $-70°$ at the epicardium and $+50°$ at the endocardium with linear dependency on the relative distance from the endocardium in between:

$$\alpha_1 = 50° - 120° d.$$

The relative distance from the endocardium was defined as:

$$d = \frac{u_{\text{endo}}}{u_{\text{epi}} + u_{\text{endo}}},$$

where u_{epi} and u_{endo} are the distances from the epicardium and endocardium, respectively. These distances were determined by solving the eikonal equation with the corresponding boundary conditions. In addition we also determined the distance from the bottom point in the apex u_{apex} by solving the corresponding eikonal equation.

The normal to a local cardial surface $\vec{\lambda_1}$ was determined by normalization of the following vector:

$$\vec{\lambda_1} \sim \nabla u_{\text{endo}} + (\nabla u_{\text{epi}} - \nabla u_{\text{endo}})\, d.$$

The line of meridian $\vec{\lambda_2}$ in each local cardial surface was found by orthogonalization of ∇u_{apex} with respect to $\vec{\lambda_1}$:

$$\vec{\lambda_2} = \frac{\nabla u_{\text{apex}} - \left(\nabla u_{\text{apex}} \cdot \vec{\lambda_1}\right) \vec{\lambda_1}}{\sqrt{1 - \left(\nabla u_{\text{apex}} \cdot \vec{\lambda_1}\right)^2}},$$

where the dot denotes the inner product. The line of latitude $\vec{\lambda_3}$ was determined by:

$$\vec{\lambda_3} = \vec{\lambda_1} \times \vec{\lambda_2},$$

where the multiplication sign denotes the vector product.

The fiber $\vec{r}$ was assumed to lie in the plane orthogonal to $\vec{\lambda_1}$ and to form the angle α_1 in between the line of latitude:

$$\vec{r} = \vec{\lambda_2} \sin\alpha_1 + \vec{\lambda_3} \cos\alpha_1.$$

References

1. Michel Goossens, Frank Mittelbach, and Alexander Samarin. *The LaTeX Companion*. Addison-Wesley, Reading, Massachusetts, 1993.

2. Streeter, D. D. J., Gross morphology and fiber geometry of the heart, Handbook of Physiology, **volume 1, pages 61 – 112 (1979)**

3. Bayer, J., , Blake, R., Plank, G., Trayanova, N., A novel rule-based algorithm for assigning myocardial fiber orientation to computational heart models, Annals of biomedical engineering, **volume 40,pages 2243–2254 (2012)**
4. Potse, M., Dubé, B., Richer, J., Vinet, A., Gulrajani, R. M., A comparison of monodomain and bidomain reaction-diffusion models for action potential propagation in the human heart, IEEE Transactions on Biomedical Engineering **volume 53 ,pages 2425–2435 (2006).**
